# Biofilm on total joint replacement materials can be reduced through electromagnetic induction heating using a portable device

**DOI:** 10.1186/s13018-024-04785-x

**Published:** 2024-05-20

**Authors:** Cordero García-Galán Enrique, Marina Medel-Plaza, John Jairo Aguilera Correa, Héctor Sarnago, Jesús Acero, José M. Burdio, Óscar Lucía, Jaime Esteban, Enrique Gómez-Barrena

**Affiliations:** 1 Dept. of Orthopaedic Surgery and Traumatology. Hospital, Universitario Príncipe de Asturias, Av Principal de la Universidad s/n, Alcalá de Henares, Madrid, 28805 Spain; 2 Dept. of Clinical Microbiology, IIS-Fundacion Jimenez Diaz, UAM. Av. Reyes Católicos 2, Madrid, 28040 Spain; 3 CIBERINFEC-CIBER de Enfermedades Infecciosas, Av. Monforte de Lemos, 3-5. Pabellón 11. Planta 0, Madrid, 28029 Spain; 4 Department of Electronic Engineering and Communications, I3A, Universidad de Zaragoza, Zaragoza, Aragon, Spain; 5 Dept of Orthopaedic Surgery and Traumatology, Hospital La Paz- IdiPAZ, Universidad Autónoma de Madrid, Madrid, Spain

**Keywords:** Biofilm, Periprosthetic, Infection, Electromagnetic, Induction

## Abstract

**Background:**

Periprosthetic joint infection is a serious complication following joint replacement. The development of bacterial biofilms bestows antibiotic resistance and restricts treatment via implant retention surgery. Electromagnetic induction heating is a novel technique for antibacterial treatment of metallic surfaces that has demonstrated in-vitro efficacy. Previous studies have always employed stationary, non-portable devices. This study aims to assess the in-vitro efficacy of induction-heating disinfection of metallic surfaces using a new Portable Disinfection System based on Induction Heating.

**Methods:**

Mature biofilms of three bacterial species: *S. epidermidis* ATCC 35,984, *S. aureus* ATCC 25,923, *E. coli* ATCC 25,922, were grown on 18 × 2 mm cylindrical coupons of Titanium-Aluminium-Vanadium (Ti6Al4V) or Cobalt-chromium-molybdenum (CoCrMo) alloys. Study intervention was induction-heating of the coupon surface up to 70ºC for 210s, performed using the Portable Disinfection System (PDSIH). Temperature was monitored using thermographic imaging. For each bacterial strain and each metallic alloy, experiments and controls were conducted in triplicate. Bacterial load was quantified through scraping and drop plate techniques. Data were evaluated using non-parametric Mann-Whitney U test for 2 group comparison. Statistical significance was fixed at *p* ≤ 0.05.

**Results:**

All bacterial strains showed a statistically significant reduction of CFU per surface area in both materials. Bacterial load reduction amounted to 0.507 and 0.602 Log10 CFU/mL for *S. aureus* on Ti6Al4V and CoCrMo respectively, 5.937 and 3.500 Log10 CFU/mL for *E. coli*, and 1.222 and 0.372 Log10 CFU/mL for *S. epidermidis*.

**Conclusions:**

Electromagnetic induction heating using PDSIH is efficacious to reduce mature biofilms of S aureus, E coli and S epidermidis growing on metallic surfaces of Ti6Al4V and CoCrMo alloys.

## Introduction

Periprosthetic joint infection is among the main complications in orthopaedic surgery, representing the second most frequent indication for replacement surgery in the knee and the fourth in the hip, with an overall incidence of about 2% [1–3]. The least aggressive among currently available surgical therapies is debridement with implant retention (DAIR). However, its indications are limited to low antibiotic-resistance germs while healing rates remain modest, currently accepted to be around 50–80% [2–4].

One of the main concerns regarding therapeutic outcomes is the ability of infecting bacteria to develop biofilm. A mature biofilm is a heterogeneous structure in which bacterial cells are embedded in a matrix. Sessile bacteria exhibit diverse genetic expressions and metabolic activities, biofilm-specific expression of antibiotic-resistance genes, and enhanced horizontal gene transmission, along with structural properties that hinder antibiotic diffusion and may prevent phagocytosis [2, 4, 5]. Altogether, these features entail a significant resistance to most current antibiotic therapies and severely limit the indications for a relatively conservative treatment such as DAIR.

Consequently, further disinfection methods are being developed besides those included in the DAIR technique (debridement, rinsing with antiseptic solutions, mechanical scrubbing, dilution, and prolonged antibiotic treatment), under the hypothesis that they could effectively enhance healing rates as a complement to current therapies. These include extrinsic physical agents such as photodynamic therapy, sonication, high-energy plasma treatment, or electric pulses [6]. In particular, heat treatment is one of the most employed methods worldwide, both in sanitary applications and in food industry, in the form of dry heat, wet heat (vapor disinfection) or pasteurization. Although dehydrated biofilms growing on dry surfaces subjected to periodic cleaning demonstrated greater resistance to dry heat treatment, hydrated biofilms that we would expect in the context of an in vivo joint infection are proven to be susceptible to heat disinfection with temperatures as low as 60ºC [7].

Electromagnetic induction heating is a recently developed technique which exploits electromagnetic induction to selectively administer heat to metallic surfaces in a contactless fashion. Thus, it allows for an antibacterial treatment with a mechanism of action different to that of chemical antiseptics and antibiotics to which bacterial biofilms present tolerance [8, 9]. In a 2017 paper, Pijls et al. demonstrated the efficacy of this technique over planktonic forms of *Staphylococcus epidermidis*, *Staphylococcus aureus*, *Pseudomonas aeruginosa*, *Bacillus cereus*, and *Candida albicans* [9]. Later, the same group published their first results over mature hydrated biofilm of *S epidermidis* [10], where a 6.7-log reduction was achieved after induction heating alone up to 60ºC for 3.5 min. Further published results explore the combined effect of induction heating and antibiotics (vancomycin, rifampicin, cefuroxime, ciprofloxacin, amoxicillin, flucloxacillin) and mechanical cleaning [11, 12].

Our group has developed a Portable Disinfection System based on Induction Heating (PDSIH, Patent solicitude EP22382889.8), designed to be feasible for its use in an operating room, unlike other devices used in previous studies.

The objective of this study is to assess the in-vitro efficacy of contactless induction-heating based disinfection of metallic surfaces using the new PDSIH over mature bacterial biofilms.

## Materials and methods

Three strains of bacterial species frequently found as arthroplasty infectious agents were selected: *S epidermidis* ATCC 35,984, *S aureus* ATCC 25,923, and *Escherichia coli* ATCC 25,922. Mature biofilms were grown on metallic coupons of two alloys commonly present in orthopaedic implants: Titanium-Aluminium-Vanadium (Ti6Al4V), and Cobalt-Chromium-Molybdenum (CoCrMo). The coupon surfaces were heated up to 70ºC for 210s with the Portable Disinfection System (PDSIH) in the experimental group, and bacterial loads were quantified and compared to assess the antibacterial efficacy.

### Study design

Experiments were conducted in triplicate: for each bacterial strain and each metallic alloy, 6 coupons were prepared. Induction-heating treatment was applied to 3 of each, while the remaining 3 coupons served as a control group. Bacterial populations on coupons belonging to the control group were incubated, rinsed with saline solutions (SS) (B. Braun, Germany), and quantified with identical procedures, but were maintained at room temperature at all times.

### Biofilm development

All bacterial strains were kept frozen at -80ºC until the experiments were performed. Each of them was cultured for 24 h on tryptic soy agar with 5% sheep blood (bioMérieux, France) at 37ºC with 5% CO_2_. After checking cultures for purity, a 0.5 McFarland turbidity suspension was prepared in SS. A 1:100 dilution of this solution (10^6^ CFU/ml) was then prepared in brain-heart infusion (BHI) with 2% glucose as a biofilm-inductor growth medium.

Biofilm was grown on cylindrical metallic coupons of two alloys: Ti6Al4V and CoCrMo. These coupons were polished discs of 18 mm in diameter and 2 mm width, according to the method described by Martínez-Pérez et al. [13].

To initiate biofilm formation, sterile polystyrene well plates (Thermo Fisher Scientific, USA) were loaded with discs. Then, 5 mL of BHI suspension from each strain was inoculated into respective wells. The plates were subsequently incubated at 37 °C with a 5% CO_2_ atmosphere for 24 h to facilitate biofilm development. After incubation, discs were washed three times with SS (B. Braun, Germany) to eliminate planktonic forms.

### Induction heating treatment

Study intervention consisted of heating metallic surfaces up to 70ºC for 210s using the Portable Disinfection System based on Induction Heating (PDSIH, Patent solicitude EP22382889.8). This device comprises a coil, an AC-DC (alternate current to direct current) converter connected to the grid, a DC-AC (direct current to alternate current) power electronic stage connected to the induction coil, the induction coil used to create the electromagnetic field that generates the heat, and a DC power supply stage connected to the DC-AC power electronic stage that provides auxiliar power. These elements are housed inside a sealable insulation housing. The insulation housing further comprises a sterilizable, electrically insulating, thermally conductive material, which makes it easily sterilizable, while also avoiding electrical risks for the user and providing effective thermal dissipation. The system has a size and a shape adapted to be used ergonomically with one hand. Moreover, the position of the induction coil is arranged at one end of the system body. In this way, the user can point the coil directly and in an easy way towards an object to be heated, thus allowing for an intuitive use of segmental induction heating suitable for complex implant geometries and compatible with usage during a surgical procedure. The output power of the power converter can be controlled through a potentiometer.

After rinsing with saline solution, each disc was placed on a Petri dish at lab temperature. PDISH was held static 11.5 mm over the surface of the disc, with the potentiometer knob fixed at 100% power. An 8s pulse was administered for initial heating up to 70ºC. Posteriorly, 1s pulses spaced with 5s cooling intervals were applied to keep surface temperature at 70ºC for 3.5 min (210s). Intervals between pulses were varied when needed to maintain surface temperature between 70ºC and 80ºC. Surface temperature was continuously monitored with a thermographic camera (Fluke® TiS75+) with emissivity fixed as 0.22. This value had been experimentally determined previously, by thermographic measurement of the coupon surfaces at known temperatures monitored with conventional digital thermometer (ThermoPro TP02S).

### Bacterial quantification

After heating, bacterial load on the upper surface of each disc was quantified by scraping. Upper surfaces were scraped with sterile wooden depressors, which were posteriorly sonicated in 10mL SS in a 50mL FalconTM conic tube (Thermo Fisher Scientific, USA), with a low power ultrasonic bath sonicator Ultrasons-H 3,000,840 (J P Selecta, Spain) at 22ºC for 5 min [14]. This sonicated fluid was 1:10 serially diluted with SS, and viable bacteria were quantified with drop plate technique [15].

### Data analysis

Statistical analysis was performed with GraphPad Prism 8.0.1 software (GraphPad Software 2018, San Diego, CA, USA). Data were evaluated using non-parametric unilateral Mann-Whitney U test for 2 group comparison. Statistical significance was fixed at *p* ≤ 0.05.

## Results

All bacterial strains showed a statistically significant reduction of CFU per surface area in both materials.

**Fig. 1:**
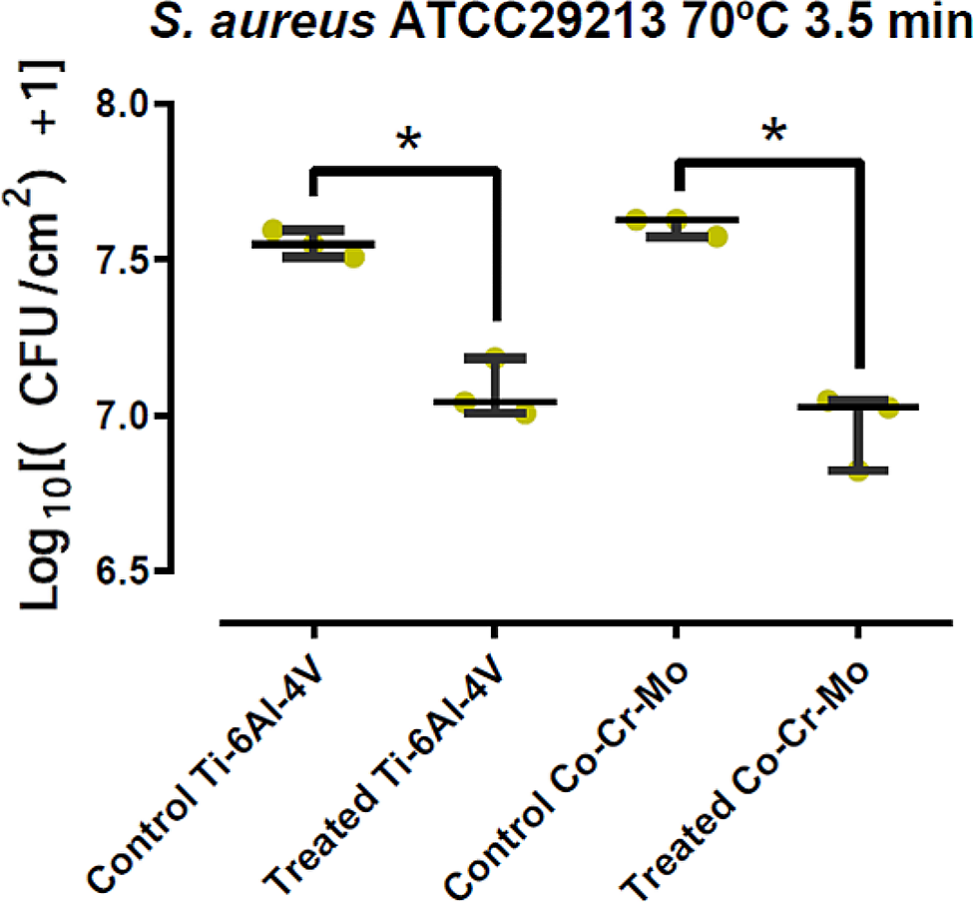
Comparative results for *S.aureus* in both materials, box-and-whisker plot

*S aureus* ATCC29213 growing on Ti6Al4V coupons presented a median (P25 – P75) of 7.549 (7.528–7.571) Log10 CFU/mL of sonicated fluid in the control group, and of 7.042 (7.024–7.112) Log10 CFU/mL in the discs treated with induction heating. This entails a 0.507 Log10 CFU/mL difference, which represents a 68.89% reduction in bacterial load with *p* < 0.05. When growing on CoCrMo alloy coupons, control group presented a 7.627 (7.600–7.627) Log10 CFU/mL median, whereas in treated coupons it was 7.025 (6.923–7.036) Log10 CFU/mL, with a 0.602 Log10 CFU/mL difference or a 75.00% reduction, *p* < 0.05. Results are summarized in the following table and plotted in Fig. 1.

**Table 1:** Bacterial quantifications for *S. aureus* on both materials, treated vs. control discs

S. aureus ATCC 29,213						
	Ti-6Al-4 V					
	**Control** [Log10 UFC/mL]		**Treated** [Log10 UFC/mL]		**Difference** [Log10 UFC/mL]	**Percent**
**Median**	7.5486080		7.0415230		0.5070850	68.889
**P25 - P75**	7.528	7.571	7.024	7.112		
	**Co-Cr-Mo**					
	**Control** [Log10 UFC/mL]		**Treated** [Log10 UFC/mL]		**Difference** [Log10 UFC/mL]	**Percent**
**Median**	7.6265840		7.0245240		0.6020600	75.000
**P25 - P75**	7.600	7.627	6.923	7.036		

**Fig. 2:**
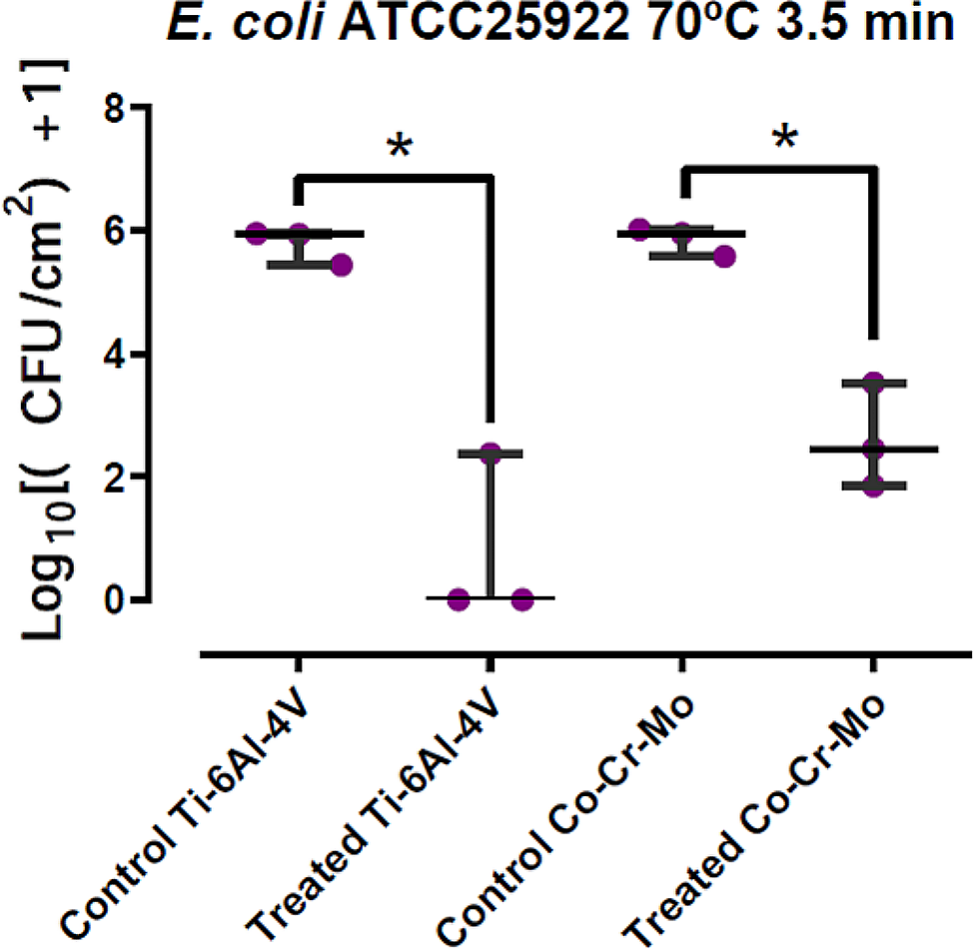
Comparative results for *E. coli* in both materials, box-and-whisker plot

*E. coli* ATCC 25,922 growing on Ti6Al4V coupons presented a median (P25 – P75) of 5.937 (5.688–5.945) Log10 CFU/mL of sonicated fluid in the control group. Viable bacteria were only present on one of the discs in the induction-heating treated group, accounting for 2.374 Log10 CFU/mL. This entails a 5.937 Log10 CFU/mL median difference, which represents a 99.99% reduction in bacterial load with *p* < 0.05. When growing on CoCrMo alloy coupons, control group presented a 5.952 (5.767–5.985) Log10 CFU/mL median, whereas the median in treated coupons was 2.452 (2.153–2.986) Log10 CFU/mL, involving a 3.500 Log10 CFU/mL difference or a 99.97% reduction *p* < 0.05. Results are summarized in the following table and plotted in Fig. 2.

**Table 2:** Bacterial quantifications for *E. coli* on both materials, treated vs. control discs

E. coli ATCC 25,922						
	Ti-6Al-4 V					
	**Control** [Log10 UFC/mL]		**Treated** [Log10 UFC/mL]		**Difference** [Log10 UFC/mL]	**Percent**
**Median**	5.9367880		0.0000000		5.9367880	99.999
**P25 - P75**	5.688	5.945	0.000	1.187		
	**Co-Cr-Mo**					
	**Control** [Log10 UFC/mL]		**Treated** [Log10 UFC/mL]		**Difference** [Log10 UFC/mL]	**Percent**
**Median**	5.9522370		2.4520290		3.5002080	99.968
**P25 - P75**	5.767	5.985	2.153	2.986		

**Fig. 3:**
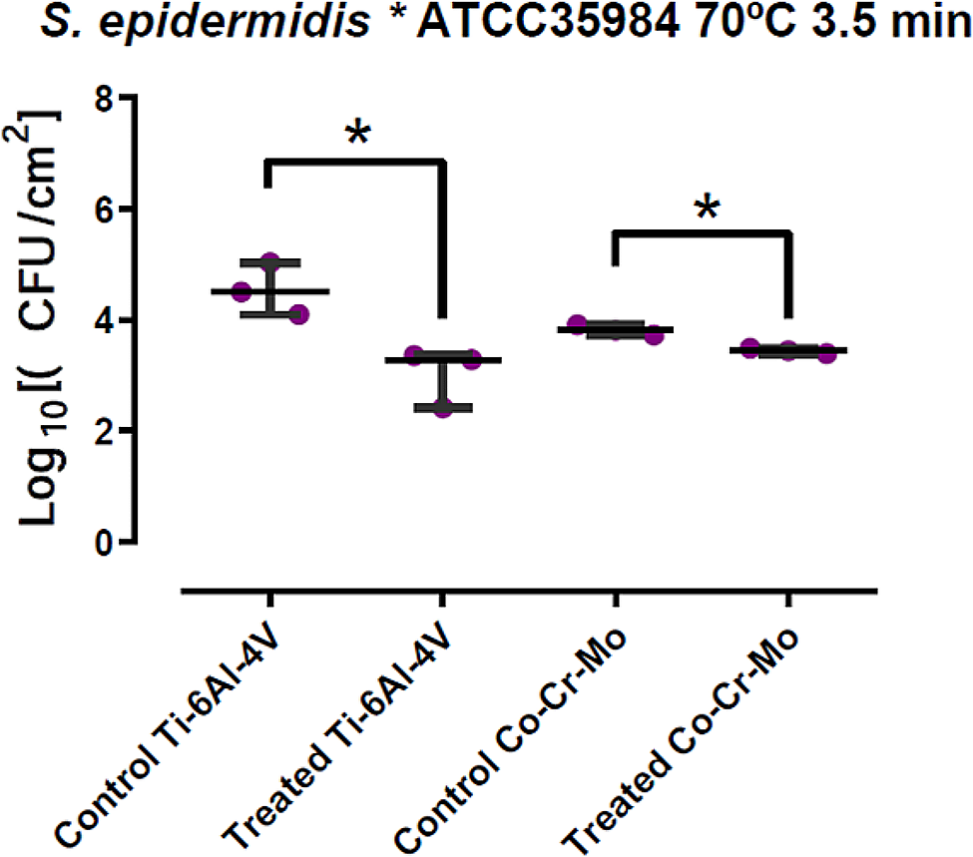
Comparative results for *S. epidermidis* in both materials, box-and-whisker plot

*S. epidermidis* ATCC 35,984 growing on Ti6Al4V coupons presented a median (P25 – P75) of 4.505 (4.303–4.769) Log10 CFU/mL of sonicated fluid in the control group, and of 3.283301229 (2.849–3.323) Log10 CFU/mL in the discs treated with induction heating. This entails a 1.222 Log10 CFU/mL difference, which represents a 94.00% reduction in bacterial load with *p* < 0.05. When growing on CoCrMo alloy coupons, control group presented a 3.820 (3.776–3.867) Log10 CFU/mL median, whereas in treated coupons it was 3.447 (3.421–3.469) Log10 CFU/mL, with a 0.372 Log10 CFU/mL difference or a 57.58% reduction, *p* < 0.05. Results are summarized in the following table and plotted in Fig. 3.

**Table 3:** Bacterial quantifications for *S. epidermidis* on both materials, treated vs. control discs

S. epidermidis ATCC 35,984						
	Ti-6Al-4 V					
	**Control** [Log10 UFC/mL]		**Treated** [Log10 UFC/mL]		**Difference** [Log10 UFC/mL]	**Percent**
**Median**	4.5051500		3.2833012		1.2218487	94.000
**P25 - P75**	4.303	4.769	2.849	3.323		
	**Co-Cr-Mo**					
	**Control** [Log10 UFC/mL]		**Treated** [Log10 UFC/mL]		**Difference** [Log10 UFC/mL]	**Percent**
**Median**	3.8195439		3.4471580		0.3723859	57.576
**P25 - P75**	3.776	3.867	3.421	3.469		

## Discussion

The main goal of this study, *id est* to demonstrate antibacterial efficacy of induction-heating treatment, was successfully accomplished as shown in our results. This serves not only to corroborate previously published results regarding induction-heating disinfection [8–12], but also to validate the newly developed PDSIH, which presents as a feasible option for operating room conditions and will thus allow for translation to in vivo settings in further studies. To our knowledge, all previous experiments assessing electromagnetic induction heating efficacy have been performed using stationary induction plates or coils [8–12]. This allows for a reproducible and easy-to-monitor technique in the lab environment, but hardly translates to the versatility, handling facility and hygienical conditions needed for further clinical applications, especially when considering the protocolized environment of the operating room.

Also, all the experiments in this study were performed with a validated model of mature biofilm on frequently used metallic alloys, and selected bacterial species are amongst the most common agents of human prosthetic joint infections [5]. Altogether, it represents one step further towards translation of this novel technique into clinical practice.

One main concern about our results is the quantitative difference with previously reported results by Pijls et al. regarding induction heating applied to mature biofilm of *S. epidermidis* on Ti6Al4V [10]. In their study, the effect was characterized for different temperatures ranging from 50ºC to 90ºC, and bacterial load reduction continuously increased. With the 3.5-minute, 70ºC induction-heating-only protocol 24-hour-biofilm, bacterial load reduction amounted to 5.8 Log10, while our study only demonstrated a 1.22 Log10 CFU/mL reduction. Possible explanations include the biological variability between the *S. epidermidis* ATCC 35,984 strain from our study, and ATCC 14,990 used by Pijls et al., unaccounted-for differences in the biofilm development technique, or also the different accuracy of the heating protocol administration and thermal image monitoring: whereas Pijls et al. employed a static induction heater placed under the sample, which was automatically controlled by an infra-red temperature sensor monitorization system and updated four times per second, we opted for a manual, less-accurate protocol, closer to that of future in vivo studies within our project. Precise quantitative results, however, should be taken with caution at this stage, for their relative magnitude and significance in the complexity of an in vivo joint infection setting is yet unknown.

In our study, a different quantitative response is insinuated between microorganisms: *E coli* showed the greatest effect, with a 5.94 and a 3.5 Log10 CFU/mL reduction on Ti6Al4V and CoCrMo respectively, whereas *S aureus* and *S epidermidis* presented less than 1.3 Log10 CFU/mL reduction on both materials. This variation could be explained by the fact that Gram-positive bacteria develop thicker, more hydrated biofilms than those of Gram-negative bacteria, which may have an effect on thermal diffusion throughout biofilm matrix. To our best knowledge, all previous studies on induction-heating on mature biofilms were performed using Gram-positive bacteria, and this result has not been reported previously.

Differences between metallic alloys may be suggested by the data, particularly in the case of *E coli*, which showed an apparently greater effect on Ti6Al4V than on CoCrMo. This may be due to the fact that no viable bacteria could be accounted for in 2 of the Ti6Al4V discs. However, the disc that did present quantifiable CFUs was well inside the range of its CoCrMo counterparts. Differences between biomaterials could not be consistently accounted for in this study.

One of the main limitations of this study is the lack of accuracy in thermal monitoring of the coupon surfaces. As stated above, the selected induction-heating protocol using PDSIH, as well as the thermographic monitoring method, were purposely selected for their feasibility for in vivo settings, and their validation was the main goal of this study. Another limitation is the fact that quantification of viable bacteria in the *S epidermidis* strain was low compared to other species, even in the control group, in both materials. This could have reduced the observable effect of the induction-heating treatment. However, bacterial load measurements are consistent between the discs, and it did not affect the statistical significance of this reduction. Finally, the simplicity of our study design can be considered as a further limitation: a full comprehension of the relative magnitude and clinical feasibility of induction-heating disinfection will require a multidimensional analysis of the isolated and combined effects of induction-heating and other techniques, such as mechanical cleaning and antibiotic therapy; the first results on this regard have been published in recent years [11, 12]. Furthermore, this study only assessed the effect of induction heating on 3 bacterial strains, while many other species can be less frequently involved in prosthetic joint infection, and induction-heating effect on them cannot be extrapolated yet. Further experiments will be needed to consistently determine the actual effectiveness of induction heating relative to other techniques, as well as possible synergistic effects, in the more complex setting of an in vivo model of joint infection, and to assess the efficacy of this technique on a wider range of microorganisms.

## Conclusions

Electromagnetic induction heating using PDSIH is efficacious in vitro to reduce mature biofilms of *S aureus*, *E coli* and *S epidermidis* growing on metallic surfaces of Ti6Al4V and CoCrMo alloys.

## Data Availability

No datasets were generated or analysed during the current study.

## Abbreviations

ATCC: American Type Culture Collection
DAIR: Debridement and implant retention
PDSIH: Portable Disinfection System based on Induction Heating
DC-AC: Direct current to alternate current
Ti6Al4V: Titanium-Aluminium-Vanadium
CoCrMo: Cobalt-Chromium-Molybdenum
SS: Saline solution
BHI: Brain-heart infusion
CFU: Colony-forming units
